# The Use and Significance of a Research Networking System

**DOI:** 10.2196/jmir.3137

**Published:** 2014-02-07

**Authors:** Maninder Kahlon, Leslie Yuan, John Daigre, Eric Meeks, Katie Nelson, Cynthia Piontkowski, Katja Reuter, Rachael Sak, Brian Turner, Griffin M Weber, Anirvan Chatterjee

**Affiliations:** ^1^University of California, San FranciscoClinical & Translational Science InstituteSan Francisco, CAUnited States; ^2^University of Southern CaliforniaSouthern California Clinical & Translational Science InstituteLos Angeles, CAUnited States; ^3^Harvard Medical SchoolHarvard UniversityBoston, MAUnited States

**Keywords:** social networking, search engine, information seeking behavior, interprofessional relations, multidisciplinary communication

## Abstract

**Background:**

Universities have begun deploying public Internet systems that allow for easy search of their experts, expertise, and intellectual networks. Deployed first in biomedical schools but now being implemented more broadly, the initial motivator of these research networking systems was to enable easier identification of collaborators and enable the development of teams for research.

**Objective:**

The intent of the study was to provide the first description of the usage of an institutional research “social networking” system or research networking system (RNS).

**Methods:**

Number of visits, visitor location and type, referral source, depth of visit, search terms, and click paths were derived from 2.5 years of Web analytics data. Feedback from a pop-up survey presented to users over 15 months was summarized.

**Results:**

RNSs automatically generate and display profiles and networks of researchers. Within 2.5 years, the RNS at the University of California, San Francisco (UCSF) achieved one-seventh of the monthly visit rate of the main longstanding university website, with an increasing trend. Visitors came from diverse locations beyond the institution. Close to 75% (74.78%, 208,304/278,570) came via a public search engine and 84.0% (210 out of a sample of 250) of these queried an individual’s name that took them directly to the relevant profile page. In addition, 20.90% (214 of 1024) visits went beyond the page related to a person of interest to explore related researchers and topics through the novel and networked information provided by the tool. At the end of the period analyzed, more than 2000 visits per month traversed 5 or more links into related people and topics. One-third of visits came from returning visitors who were significantly more likely to continue to explore networked people and topics (*P*<.001). Responses to an online survey suggest a broad range of benefits of using the RNS in supporting the research and clinical mission.

**Conclusions:**

Returning visitors in an ever-increasing pool of visitors to an RNS are among those that display behavior consistent with using the tool to identify new collaborators or research topics. Through direct user feedback we know that some visits do result in research-enhancing outcomes, although we cannot address the scale of impact. With the rapid pace of acquiring visitors searching for individual names, the RNS is evolving into a new kind of gateway for the university.

## Introduction

New tools are enabling the search and discovery of researchers, their expertise, intellectual output, and professional networks. These Web-based applications mine a variety of data sources to automatically generate searchable profiles and expose existing networks of collaborators. In addition to large-scale commercial services [1], universities have begun to deploy such systems locally. Medical schools were early adopters, motivated by the promise of these tools in enabling the development of diverse research teams to meet the evolving emphasis of funders and the demands of translational science [2,3]. Momentum for these research “social networking” systems or research networking systems (RNSs) has continued to build; a recent pilot of a federated search of expertise counted 57 institutional participants [4,5]. Although they originated in biomedicine, the systems are now being extended to represent diverse institutional portfolios of research expertise [6,7].

RNSs deployed at medical schools can be contextualized against industry deployments of “expertise location” and “social networking” systems [8]. Like expertise location systems, RNSs enable the discovery of individuals based on their expertise using the automated generation of rich searchable profiles. Like enterprise social networking systems, RNSs allow for the search and browsing of networks of people and topics. Such systems are being deployed internally by large intellectual capital-driven companies (such as IBM [9] or Deloitte Consulting [10]) for reasons similar to those motivating the deployment of RNSs in academic settings (ie, to facilitate a better understanding of who’s doing what and to enable knowledge sharing and team building [11]). But, RNSs are different in one significant way. Because they support the academic mission and because research increasingly requires collaboration beyond institutional boundaries [12], they are deployed as primarily public systems. As a result, unlike closed enterprise systems, a public RNS exposes rich content about the people in an institution to the broadest possible audience.

With an increasing number of RNSs deployed in academic biomedicine, there is now a focus on understanding how these tools are used. In industry, studies show that such tools are used predominantly to search for people [13] including those outside known circles of colleagues [9,14]. This is in contrast to findings from studies of users of Facebook where evidence suggests that users primarily reinforce existing networks, though this has only been studied in student populations [15,16]. In the academic setting, only one study reports use of a small private RNS showing that visitors who do log on spend more time in their session than comparable benchmarks for time spent on Google sessions [17]. In addition, several studies have documented needs and requirements for such systems to enable collaboration in science [18-20]. Outside of an organizational setting, LinkedIn’s membership grew to more than 200 million members in 2013 [21], providing general testament to the perceived value of professional networking tools.

In 2010, the University of California, San Francisco (UCSF) became the first external adopter of an open source RNS called “Profiles Research Networking Software” [22]. After several years of deployment, we present a description of the usage of this publicly accessible RNS that exposes rich content about biomedical researchers at the institution to the broadest possible audience. Our objective is to provide the first description of the degree of usage of a public institutional research networking system and to identify sources of visitors, rate of engagement (visitors returning), degree of engagement (depth of visit), and outcomes as reported by users. We also wanted to assess evidence of usage of the more unique aspects of the RNS that allow users to view related people and concepts, exposing them to the connections that might induce identification of new collaborators or research themes. And finally, we were curious about how the introduction of a public social networking system might transform access to a university’s primary intellectual capital—its people.

## Methods

### Ethics: Human Subjects Research

The study plan was submitted to the UCSF Institutional Review Board (Human Research Protection Program) and was determined not to be human subjects research.

### UCSF Profiles Research Networking System

**Figure 1:**
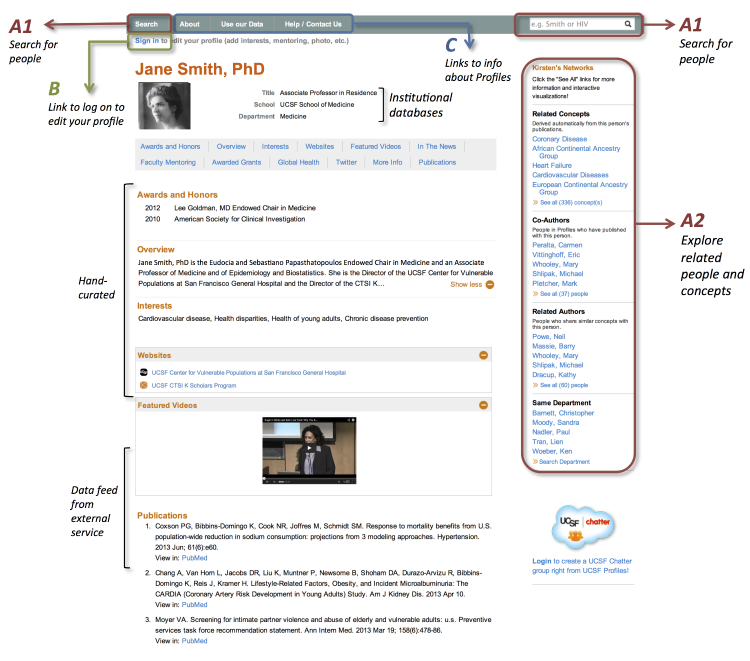
Screenshot of profile in UCSF Profiles. Subareas A, B, C denote 3 sets of links that when clicked through count as an additional page on site. Subareas A1 and A2 denote links connecting visitors to related people and topics.

A screenshot of the UCSF Profiles RNS is shown in Figure 1. Multiple sources of data populate the contents of individual profile pages. These sources include publication feeds from the National Library of Medicine’s PubMed, disambiguated to match author names to institutional sources of information [22]. UCSF Profiles extends the open source Profiles RNS in multiple ways, including integrating relevant commercial services such as YouTube and SlideShare.

### Naming Conventions

We abbreviate the RNS “UCSF Profiles” to “Profiles”. We also use the term “profile” without capitalization to denote individual pages describing researchers. Although we describe the users and usage of an RNS, we refer to those who use the site as “visitors” to minimize confusion between our description of those who visit the site versus those who own profiles on the site and can log in to access additional owner-related functions.

### Time Period of Analysis

To understand visitors to Profiles and how they evolved over time, we analyzed data from December 2009 (the beta launch of Profiles) through December 2012. We primarily present time course data for the 28 months from the public launch in September 2010 through December 2012 and summary data from the last 6 months analyzed (Months 23-28), from July 2012 through December 2012.

### Population Represented in Profiles

Individuals were added mostly en masse and offered the option to opt-out of the system (7 did), or invited to opt-in if their relationship with the university was less formal or if otherwise deemed appropriate (for example, faculty whose primary affiliation is with another institution but have “without-salary” appointments at UCSF). A total of 7297 profiles were created from the launch date to December 31, 2012, at which point there were 5928 active profiles. The difference represents people no longer at UCSF and the 7 who chose to opt out. Individuals added to Profiles by December 2012 included 2691 full time faculty, 356 without-salary faculty, 386 instructors, 2079 trainees (postdoctoral scholars, residents, and fellows), and 1785 other university leaders, researchers, and staff.

### Web Analytics

Website usage was measured and analyzed primarily using Google Analytics, which is an industry standard Web analytics measurement framework [23], used by 51% of Fortune 500 companies [24] and 66% of the top 10,000 websites online [25]. We chose Google Analytics for its long-term support, and extensive data filtering and segmentation capabilities—reasons why it has begun to be used for other academic sites as well [26].

We used terminology consistent with that used by Google. We filtered out IP addresses associated with our internal office to remove the effect of internal testing and usage. “Visit” indicated a series of one or more interactions with the website, separated by gaps of no more than 30 minutes between subsequent interactions. “Pages/Visit” represented the number of individual Web pages accessed over the course of a visit. “Time on Site” meant the length of time between the first and last interaction during a visit (time on site is ignored for visits of only one page). “Visitor Location” indicated a visitor’s organizational and/or geographic location as inferred by performing geolocation and network ownership analysis of a user’s IP address. We divided locations into five non-overlapping categories: UCSF, San Francisco, California, US, and World.

“UCSF” included visitors who originated from an IP address associated with the UCSF internal network; 99.73% (114,944/115,258) of these visits originated from facilities in the City of San Francisco. This underestimates UCSF visitors because it excludes some UCSF sites (eg, San Francisco Veterans Administration Hospital, the San Francisco General Hospital, and the Gladstone Institutes) and those using mobile devices or working off-site without a VPN. To help us understand the degree to which we underestimated UCSF visits, we analyzed IP addresses of those who opened emails targeted specifically to UCSF employees (using the tool MailChimp). Of 615 opened emails, 344 (55.9%) were opened from campus IP addresses and 271 (44.1%) from other external IP addresses. Though it may be more common for mobile visitors to read email than access websites, this nevertheless suggests that visits from the UCSF campus network reflect only a portion of visits from all UCSF personnel, and the actual percentages could be up to 79% higher than the numbers we see coming from the campus network. In this paper, however, when we refer to UCSF visitors, we mean only those unambiguously located with the campus network.

“San Francisco” included visitors who originated from the City of San Francisco, other than those from the UCSF internal network. We expect that this included almost all of the remaining UCSF sites that were not otherwise covered by the UCSF network, though their contributions to overall visits are small. San Francisco is also the location most likely to capture traffic from patients of the UCSF Medical Center.

“California” included visitors who originated from California other than those from either San Francisco or the UCSF internal network. This too included some UCSF-related traffic, since many researchers and staff live in the greater Bay Area outside of San Francisco. “US” included visitors who originated from the United States outside of California, and “World” included visitors who originated from sites outside the United States.

Under “Referring Source”, terms included (1) “Search”, visits from search engines (eg, Google, Bing, Yahoo, UCSF.edu search engine), (2) “Website Referrals”, visits from other websites, excluding traffic via search engines, and (3) “Direct or Unknown”, visits from sources that cannot be programmatically identified (eg, user typing in “profiles.ucsf.edu” on their Web browser, clicking a browser bookmark, clicking a link in a desktop email or Twitter client, or following a link from websites that use the secure HTTPS protocol to the non-secure Profiles website). For “New and Returning Visits”, returning visits are those sessions (visits) where the visitor is recognized as having visited the site before via the presence of cookies. Because of the reliance on cookies, this is generally an underestimate of the true value.

### Categories of Visitor Actions

We report on “Depth of Visit”, which can be measured as time on the site or as pages per visit (pages/visit). We used pages/visit since that most directly addresses the next steps visitors take after viewing a profile page. A one-page visit might mean that the visitor arrived on a profile page and then stopped using Profiles or clicked a link to some other website (such as PubMed) without returning in the next 30 minutes. Any visit that lasts for 2 or more pages occurs because a visitor clicks on one of three types of links from a profile page. These three categories of links are depicted in Figure 1. The first set of links (A) reveals related researchers or topics either through reinitiating a search (A1) or by clicking on a name, topic, or link that expands to related names or topics (A2). The second link (B) enables editing of one’s own UCSF profile. The third set of links (C) describes Profiles.

### Feedback Survey Analysis

Between September 3, 2011 and January 31, 2013, site visitors were asked, “How has Profiles helped you?” The survey appeared on all Profiles pages to all visitors (unless the visitor chose to minimize the survey’s inline pop-up), and 475 comments were submitted. Of these, 5 comments were deleted for having no content or email address, and 1 was deleted as it came from a Profiles developer. The remaining 469 responses were binned into seven categories based on the content and submitter’s email address (if available): (1) Enables Research, (2) Provides Background Information for Clinical Care, (3) Provides Contact Information for Clinical Care, (4) Generally Positive, (5) Negative, (6) Other, and (7) Spam.

### Statistics

We assessed whether depth of usage of Profiles (measured as pages per visit) differed based on whether a visit was a first-time or returning visit. We also assessed whether UCSF visitors differed in their behavior from other visitors. Unfortunately, Google Analytics does not provide access to raw data describing depth of visit (pages/visit) for each individual visit and as a result statistics were performed on daily averages for each category measured. Daily averages were analyzed for 6 months from July 1 through December 31, 2012, resulting in n=184 days for which average daily depth of visit was calculated for new and returning visitors. A standardization approach was used to control for varying “n” contributing to daily averages. Two-factor analysis of variance (ANOVA) with multiple comparisons was performed on the data. The 2x5 ANOVA assessed whether there was an interaction effect of visitor status (new or returning) with location (UCSF, San Francisco, California, USA, World) in explaining depth of usage of Profiles. All tests were two-sided and a statistical comparison or model was considered significant if *P*<.05. All analyses were performed in Stata v.12.

## Results

### External Visitors Contribute to Rapid Increases in Visits

**Figure 2:**
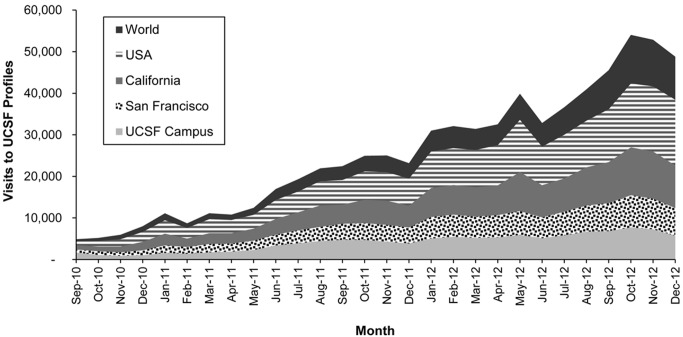
Location of visitors to Profiles for the 28 months since launch.

Figure 2 describes the pace at which visits to Profiles increased over the 28-month period since launch (September 2010-December 2012). The figure also depicts the institutional and/or geographical location of visitors, ranging from UCSF campus to visitors coming from outside the United States. In the 28 months following launch, traffic to Profiles increased both from on-campus and off-campus sources to an average of 46,000 visits per month as measured for the last 6 months of data analyzed, from July to December 2012. Over this 6-month period, out of a total of 278,570 visits, 40,140 (14.41%) of visits came from UCSF and 238,640 (85.67%) from outside UCSF. As we note in the Methods section, the true percentage of visits from UCSF may be up to 80% higher. Out of the total 278,570 visits, those from outside UCSF can be broken down as 40,438 (14.51%) from San Francisco, 60,256 (21.63%) from California, 81,238 (29.16%) from the United States, and 56,708 (20.36%) from outside the United States. Each category excludes the prior. Visits have continued to increase rapidly; in January 2014, the site received over 85,930 visits, and the last week of January saw more than 3000 visits per weekday.

### Search Trumps All Other Sources of Visits

**Figure 3:**
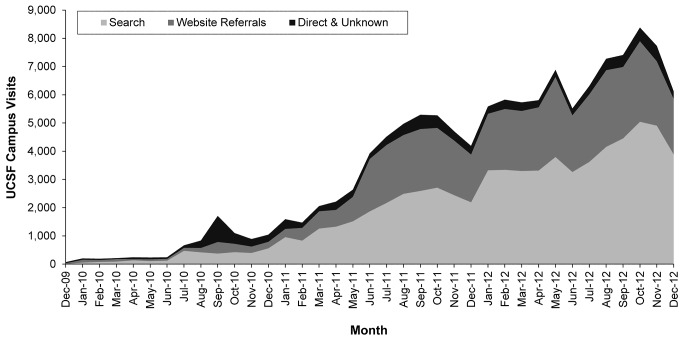
Time course of growth in traffic from various referral sources (peak in direct traffic in September 2010 coincides with launch activities at UCSF).

We assessed how visitors arrived at Profiles by analyzing the referring sources of traffic. From July to December 2012 (Months 23-28), search traffic accounted for 208,304 (74.78%) of the 278,570 visits to Profiles, referrals from other websites accounted for 47,861 (17.18%), and direct or unknown sources for 21,926 (7.87%). Google dominated search traffic; 190,170 (91.29%) of the 208,304 visits from a search engine came via Google, while another 7992 (3.84%) came via the search engine on the UCSF website, which is powered by Google and uses the same underlying ranking algorithms. The remaining 10,142 (4.87%) of search-driven traffic (“Other search”) came via sources such as Bing and Yahoo. Visitors from all sources arrived on Profiles via search, but non-UCSF visitors had a greater likelihood of landing on Profiles via a search engine than UCSF visitors (80% for non-UCSF vs 63% for UCSF). The time course of visitor acquisition from search, website referrals, and direct or other sources is shown in Figure 3.

### Visitors Arrive at Profiles Predominantly by Searching for People’s Names

We analyzed 250 search queries that landed on Profiles randomly selected from the set of such queries during Months 23-28. In this sample, of all searches that sent visitors to Profiles, 210 (84.0%) included a name, with half of these only including a person’s name (42.0%, 105/250). Another large set used the name along with a reference to the university (33.2%, 83/250; eg, <name> ucsf ; <name> university of california san francisco). A smaller set searched for a name along with additional information (8.8%, 22/250; eg, <name> radiation oncology). Finally, 10.0% (25/250) used the name of a paper, and the remaining 6.0% (15/250) included a mix of the name of the university (without other information), URLs, and other data.

### Visitors Return in Increasing Numbers

The percentage of visits from returning visitors doubled in the first year (from 1203 of 7039 visits, or 17.09%, in the month of launch, September 2010, to 7235 of 21,924 visits, or 33.0%, in August 2011) but remained constant after that. But as visits continued to increase, this means that the number of visitors who returned to the site continued to increase as well. A higher proportion of UCSF visits included returning visitors (26,758 of 40,140, or 66.66% in Months 23-28) compared to non-UCSF visits (66,105 of 238,430, or 27.73% in Months 23-28). But returning visits continued to increase in absolute numbers for both UCSF and non-UCSF visits. Returning visits are of particular interest not only because they represent a group of visitors who find the tool useful, but, as we show below, these visitors also tend to use the tool in more depth.

### Subset of Visitors Continue Beyond Profile Page to Explore Related People and Topics

**Figure 4:**
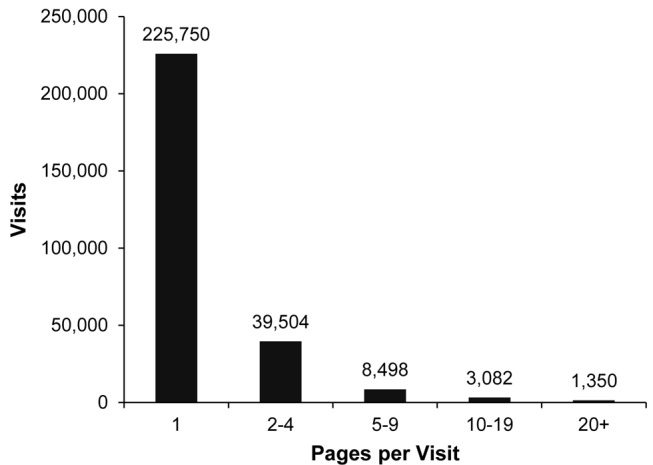
Distribution of visitors by depth of visit (pages/visit) during Months 23-28 from launch.

We analyzed the distribution of visits by depth of visit for the period from Months 23-28. As Figure 4 shows, the majority of visits during Months 23-28 resulted in the viewing of only 1 page (81.15%, 225,750/278,184) with the remaining 18.85% (52,434/278,184) extending for 2 or more pages. Visits that went 2 or more pages utilized links shown in Figure 1 in categories A, B, and C. Even though most visits were only 1 page deep, a significant number of visits included multi-page browsing. For example, in January 2013, the site received 2641 visits (or >85 visits a day) where visitors browsed the site for 5 or more pages. To provide a flavor of the average time spent on site during this period, an average 2-page visit lasted for 4 minutes and 8 seconds, a 10-page visit lasted for 12 minutes and 8 seconds, and a 20-page visit lasted for 17 minutes and 34 seconds.

To further understand the distribution of actions taken by visitors immediately after they landed on a profile page, we evaluated actions taken on the first page of 1024 visits to profile pages randomly selected from all visits during Months 23-28. The results are described in Table 1. In this sample, 76.27% (781/1024) either left the site after viewing 1 page or took a path that Google was unable to identify. Visits that left the site included those that clicked on links for individual publications. The remaining 23.73% (243/1024) would be counted as visits with 2 or more pages viewed. These can be broken down as 10.84% (111/1024) clicked to see other related people or concepts (eg, as presented by the novel networking elements of the RNS, Category 1A and B in Figure 1), 2.34% (24/1024) clicked back to the list of search results and clicked on another person, and 7.71% (79/1024) initiated a new search. A small number went on to edit their own profile (0.98%, 10/1024, Category B) or clicked on a site-wide informational link (1.86%, 19/1024, Category C).

**Table 1:** Actions taken from a profile page (n=1024).^a^

Action category	n (%)	Descriptive data
Leave (Exit)	706 (68.95)	Profile page is last page user looked at before leaving the website
**More about that person or topic’s relationships** ^b^		
	20 (1.95)	Clicks on list of all Co-Authors
	12 (1.17)	Clicks on list of all Similar People
	5 (0.49)	Clicks on list of all Keywords for that person
**Another person** ^b^		
	74 (7.23)	Clicks to another user’s profile (eg, by clicking on a Co-Author, Similar Person, etc, link)
	24 (2.34)	Clicks back to list of search results, then clicks on another person
New Search^b^	79 (7.71)	Runs a new search (eg, by entering text in mini search box)
**Site-wide navigation**		
	13 (1.27)	Clicks to homepage
	6 (0.59)	Clicks on "How Profiles Works" page
Edit Profile	10 (0.98)	Clicks on edit profile link
Unknown	75 (7.32)	Unknown or unrecorded

^a^Analysis of user behavior on the initial landing page for 1024 visits to profile pages randomly selected from all visits during Months 23-28.

^b^Paths to “networked links” that connect the initial profile page to related people or topics.

### Returning Visitors Use the Site More Deeply Than First-Time Visitors

**Figure 5:**
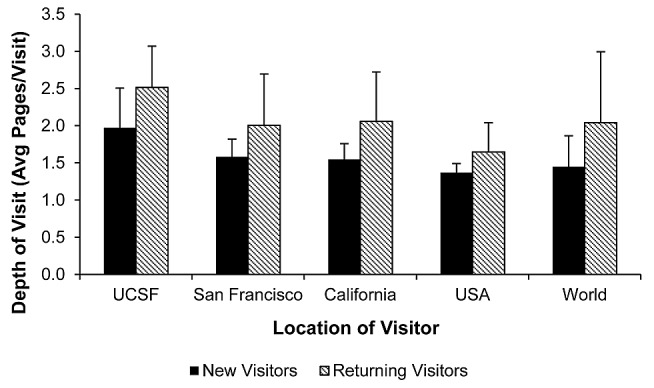
Depth of visit (pages/visit) for new and returning visitors. The figure describes visitors originating from different locations during Months 23-28 from launch. Error bars show 1 Standard Deviation.

Figure 5 shows the depth of visit as average pages/visit for new and returning visitors coming from different locations. Returning visitors with an average 2.103 pages per visit went deeper than new visitors with an average 1.496 pages per visit, regardless of the location they came from (*P*<.001, standardized mean difference of 1.099). Depth of visit also depended on the location that visitors came from with UCSF visits resulting in the deepest visits (*P*<.001). Although visit numbers were lower on weekends, the depth of visit did not differ for any day of the week.

### Users Provide Direct Feedback Revealing Broad Utility of RNS

In September 2011, we implemented an online user feedback form that asked visitors, “How has Profiles helped you?” During the 16 months after implementation (through Jan 2013), 469 submissions were received from a total of 670,000 visits, with 0.07% of all visits converted to a submission. Overall, 284 (60.5%, 284/469) comments describing positive outcomes were submitted. We found 58 (12.4%, 58/469) described how Profiles enabled research, 91 (19.4%, 91/469) described how Profiles enabled clinical care by providing contact information, 18 (3.8%, 18/469) described how Profiles enabled clinical care by providing research-related information on clinicians, and 117 (24.9%, 117/469) were positive without specific details. In addition, 118 (25.2%, 118/469) categorized as “Other” primarily requested help with the tool, 3 (0.6%, 3/469) were spam, and 64 (13.6%, 64/469) were “Negative”. Of the “Negative” comments, 29 (45%) out of 64 left negative comments reflecting their unmet expectations of a traditional directory listing (such as organization by department or sub-specialty), while 21 (33%) simply said “no”, and the remainder noted a negative comment related to technical difficulties. We show a sample of the comments, categorized as “Enabling Research”, in Textbox 1. Visitors self-reported that they found collaborators, identified new research problems, and improved administrative processes.

**Examples of user feedback that described Profile’s positive impact on research. The question, “How has Profiles helped you?” was asked on a pop-up survey automatically presented to all visitors. The survey was deployed during Months 13-29, from September 2011 through January 2013. These are 13 illustrative quotes of user feedback from the 58 user comments that were categorized as “enabling research”.**

I am hoping that it just helped me find a mentor...

Found speakers for grand rounds at SFGH pediatrics.

Great resource for finding potential research collaborators and for PhD dissertation committees.

It is wonderful to find the current publications. My faculty rarely tell me. I need it for our annual report and for some grant applications.

This profile allowed me to review the background and experience of an author from a journal article I was reading.

Research information about scientists for my biomedical foundation.

Highlights/generates awareness about science accomplishments beyond my immediate sphere of research but very stimulating and interesting.

Gives me a profile of a potential contributor to a book that is under consideration, helps to locate people in a specific field. Important service!

Making your remarkable research available to me and my translational research projects is invaluable. Thank you so very much.

Trying to find interesting colleagues to meet with during my stay at UCSF.

Quite useful in introducing UCSF colleagues to others outside the university. Also a quick way to get a feel for the interests of people I don’t know.

To find researchers with common interests.

Contact a faculty member recommended by an associate. Initiate a sponsored project.

## Discussion

### Principal Findings

Research networking systems automatically generate and publically display profiles and networks of researchers. Within 2.5 years, our system, UCSF Profiles, achieved one-seventh of the monthly visit rate of the longstanding main university website, with a continually increasing trend. Visitors came from diverse locations beyond the institution. Responses to an online survey suggest a broad range of benefits supporting the research and clinical mission. Returning visitors are among those that appear to use the system to identify new collaborators or research topics. And, with the continually increasing visits arising from public searches for individual names, UCSF Profiles is evolving into a new kind of online gateway for the university.

### Assessing and Improving the Usage of Research Networking Systems

Schleyer et al propose a comprehensive framework to advance the study of research networking systems [26] within which this work would fall under their “evaluation” axis. They define RNSs as systems that enable collaboration and develop a framework to test that specific hypothesis. In contrast, we take an empirical approach to understanding the role of an RNS at an academic institution and assess our customers and their online behavior just as a commercial vendor would. The profile page of a person of interest was the main entry point for visitors. For those that left the page (nearly 80%), we cannot distinguish between those who began a new task from those who continued to explore the original person of interest but had to leave the site (eg, by going to a publication in PubMed or clicking on a YouTube video of a talk). For those who continued on the site, almost all of the visitors (roughly 20%) clicked on links of people who were not the original person of interest but were displayed as related people (coauthors or similar people), or clicked on topics exposed by the tool, which in turn led to a new listing of people. We interpret our results as showing that at least one-fifth of visits initiated the process of exploring a related researcher or topic, someone or something the visitor had not considered in their initial search. But we cannot conclude how frequently they achieved their goal. On the other hand, visits continue to increase at a rapid pace and visitors return and stay longer in increasing numbers, suggesting a positive outcome and perceived utility for a growing segment. Specific responses to our online survey provide anecdotal evidence of impact and define a framework for systematic assessment. In addition to identifying collaborators and building teams, based on the responses, the framework should assess impact on creating efficiencies in research administration, enabling broader research functions such as mentoring, and advancing the clinical mission.

Although the initial goal of Profiles was to enable collaboration by making it easier for researchers to find partners and build teams, the most striking aspect of its deployment was how rapidly visitors were drawn from both within and outside the university. In January 2013, visits to UCSF Profiles were one-seventh of the overall visits to the established UCSF campus website and, at the time of publication, visits had caught up to almost one-fifth (18%) of that traffic. We attribute several factors to this increase in visits. As Figure 3 describes, increases in visits came both from increased website referrals and from significant increases in visitors coming from search engines. Most of the increases in visits from website referrals reflect increased numbers of UCSF visitors finding Profiles through campus websites. We initiated a high-level partnership with the University Relations office and developed a coordinated strategy to promote university faculty and research. A key element was standardizing links for faculty who were being publicized so they always pointed to their UCSF Profiles page. We also realized that the directory was a significant point of entry for the university. A partnership again allowed us to embed links to profiles in the university directory. Many other interventions, including a data federation strategy where departments could use publication and other feeds from Profiles to enhance their own websites, brought goodwill and adoption across campus, but contributed only a small proportion of visits overall. This was because, as we show, visitors from search engines dominated all sources, which in turn was enabled through a comprehensive search engine optimization strategy implemented early in the deployment of Profiles. While details of search engine optimization techniques are out of scope for this paper, broadly, we encouraged links to UCSF Profiles for a variety of on- and off-campus websites, copyedited HTML page titles and page descriptions, implemented HTML people data microformats, simplified URLs to “profiles.ucsf.edu / firstname.lastname”, implemented a sitemap, and cleaned up redirects and error page HTTP codes.

In efforts to understand the rapid pace of acquiring visitors, we also found that most visits to Profiles came from visitors searching for a name. As visits continue to increase, Profiles continues to capture more of this type of visitor. Where might these Internet users have landed had they not found Profiles? Perhaps not the primary university website—compared to more than 70% of visits arriving via search to Profiles, only 25% of visits come via search to the university website. But, most researchers have multiple affiliated websites, some official such as departmental, laboratory, or clinical, and some not, such as news articles, and some related to other organizations such as a journal or corporation. The visits being captured by Profiles were otherwise likely to be dispersed among these various possibilities. Instead, they are now being aggregated and captured by the university RNS. And, with its search-optimized interface and increasing number of personally-curated elements, the site is also becoming the link of choice for external media. When Gurpreet Dhaliwal was profiled in the *New York Times* as a masterful clinical diagnostician, the newspaper linked to his profile in UCSF Profiles [27]; a variety of other media outlets have also linked to individual profiles.

### People First: A New Gateway Exposes the Strengths of the University

An assessment of the searches that bring visitors to Profiles does not describe the universe of searches. Still, of those brought to Profiles via search engines, we found that 84% searched for individuals’ names with or without accompanying search terms including the institution’s name. But regardless of whether a visitor to Profiles is aware of the institutional affiliation of the person for whom they search, by the time they land on a profile page they are in effect being exposed to the institution. And when they continue on the site, they are introduced to the university through the unique lens of expertise and intellectual networks, not schools, departments, or administrative organization. That Internet users search for people at a university independent of their interest in the university itself makes sense even if we may not have predicted the volume of this trend. Public affairs offices have long understood this—promoting the university through news stories about individual faculty. But an RNS such as Profiles (among others [4]) is explicitly designed to promote people, their intellectual outputs, and networks at scale. Thus, an RNS presents data in a format optimized for the most ubiquitous consumer tool (search engines) delivering content optimized around consumer interests (names of individuals). As a result, the institutional RNS is evolving into a new online gateway for the university, providing a discoverable interface to the intellectual capital of the institution: its people, knowledge, and networks. With this new frame, we are reassessing the positioning of this publicly accessible enterprise system within the fabric of the university. Further integration of the RNS into the university public relations office, into the Executive Vice Chancellor and Provost’s office, and indeed into the medical center’s marketing and communications arm are relevant next steps.

## Abbreviations

ANOVA: analysis of variance
RNS: research networking system
UCSF: University of California, San Francisco
